# Rapid dissemination of alpha-synuclein seeds through neural circuits in an in-vivo prion-like seeding experiment

**DOI:** 10.1186/s40478-018-0587-0

**Published:** 2018-09-19

**Authors:** Ayami Okuzumi, Masaru Kurosawa, Taku Hatano, Masashi Takanashi, Shuuko Nojiri, Takeshi Fukuhara, Tomoyuki Yamanaka, Haruko Miyazaki, Saki Yoshinaga, Yoshiaki Furukawa, Tomomi Shimogori, Nobutaka Hattori, Nobuyuki Nukina

**Affiliations:** 1 Department of Neurology, Juntendo University Graduate School of Medicine, 2-1-1 Hongo, Bunkyo-ku, Tokyo, 113-8421 Japan; 2 Institute for Environmental and Gender-specific Medicine, Juntendo University Graduate School of Medicine, 2-1-1 Tomioka, Urayasu-shi, Chiba, 279-0021 Japan; 3 Department of Neurology Juntendo University Koshigaya Hospital, 560 Fukuroyama, Koshigaya city, Saitama, 343-0032 Japan; 4 Medical Technology Innovation Center, Clinical Research and Trial Center, Juntendo University Graduate School of Medicine, Tokyo, Japan; 5 Laboratory of Structural Neuropathology, Doshisha University Graduate School of Brain Science, 1-3 Tatara Miyakodani, Kyotanabe-shi, Kyoto, 610-0394 Japan; 6 Laboratory for Mechanistic Chemistry of Biomolecules, Department of Chemistry, Keio University, 3-14-1 Hiyoshi, Kohoku, Yokohama, 223-8522 Japan; 7 Laboratory for Molecular Mechanisms of Brain Development, RIKEN Center for Brain Science, 2-1 Hirosawa, Wako, Saitama, 351-0198 Japan

**Keywords:** Rapid dissemination, A-syn, Propagation, Callosotomy

## Abstract

Accumulating evidence suggests that the lesions of Parkinson’s disease (PD) expand due to transneuronal spreading of fibrils composed of misfolded alpha-synuclein (a-syn), over the course of 5–10 years. However, the precise mechanisms and the processes underlying the spread of these fibril seeds have not been clarified in vivo*.* Here, we investigated the speed of a-syn transmission, which has not been a focus of previous a-syn transmission experiments, and whether a-syn pathologies spread in a neural circuit–dependent manner in the mouse brain. We injected a-syn preformed fibrils (PFFs), which are seeds for the propagation of a-syn deposits, either before or after callosotomy, to disconnect bilateral hemispheric connections. In mice that underwent callosotomy before the injection, the propagation of a-syn pathology to the contralateral hemisphere was clearly reduced. In contrast, mice that underwent callosotomy 24 h after a-syn PFFs injection showed a-syn pathology similar to that seen in mice without callosotomy. These results suggest that a-syn seeds are rapidly disseminated through neuronal circuits immediately after seed injection, in a prion-like seeding experiment in vivo, although it is believed that clinical a-syn pathologies take years to spread throughout the brain. In addition, we found that botulinum toxin B blocked the transsynaptic transmission of a-syn seeds by specifically inactivating the synaptic vesicle fusion machinery. This study offers a novel concept regarding a-syn propagation, based on the Braak hypothesis, and also cautions that experimental transmission systems may be examining a unique type of transmission, which differs from the clinical disease state.

**Electronic supplementary material:**

The online version of this article (10.1186/s40478-018-0587-0) contains supplementary material, which is available to authorized users.

## Introduction

Parkinson’s disease (PD) is one of the most common neurodegenerative disorders. The primary manifestations of PD consist of movement disturbances, such as bradykinesia, tremor, and rigidity [14], while the main cellular pathological features include neuronal degeneration along with inclusions called Lewy bodies (LBs), and neuronal loss in the substantia nigra (SN) [4]. The protein alpha-synuclein (a-syn), a major component of LBs and Lewy neurites, is deposited in a phosphorylated form. These a-syn deposits are also observed in dementia with Lewy body and in multiple system atrophy [15]**.** However, the reason for the a-syn deposits to result in distinct disease phenotypes [16, 35] remains unclear.

Studies on PD brains reveal that a-syn pathologies spread to the brainstem from the olfactory bulb and the enteric vagus nerve during the first several years of the disease. As the disease progresses, the pathology spreads to other brain areas, over the course of 5–10 years [4, 5, 18]. Therefore, the intracerebral growth of a-syn pathologies is considered to be the underlying mechanism for disease progression, and the localization and abundance of a-syn deposits tend to correlate with the clinical symptoms. Consequently, a deeper understanding regarding the spread of a-syn deposits is needed, in order to clarify the mechanisms underlying disease progression. In recent years, both in vitro and in vivo studies have indicated that pathological a-syn spreads to adjacent cells and anatomically connected areas of the brain. In addition, this spreading has been reported to occur in a prion-like manner, upon the injection of recombinant a-syn including monomers, oligomers, fibrils or insoluble a-syn derived from diseased brain, or spinal cord homogenates into the brain of a-syn overexpressing/wild-type rodents [17, 19, 25, 26, 28, 29, 33, 35, 40–42] or primate [37, 44] and also into the intestine wall of the stomach [19]. Another report has proposed the hypothesis that these seeds induce the conversion of endogenous a-syn to the abnormal form of a-syn and its deposition, although this has not been demonstrated in vivo [32].

This observation suggests that the synaptic connections between neurons may be involved in the spread of a-syn pathologies. However, this possibility needs to be investigated experimentally. Therefore, in this study, we used a neural disconnection approach in the mouse brain, in which either callosotomy or botulinum toxin B (BoNT/B) injection was performed before and after a-syn seeds injection to examine transmission speed, and the specific neural circuits through which a-syn pathologies spread. Although it is believed that clinical a-syn pathologies take years to spread throughout the brain, this study showed that seeds exhibit rapid dissemination throughout the brain via neural networks, within 24 h. This offers a novel concept to add to the existing discussion of disease progression in the brain, and also cautions that artificial experimental transmission systems may be examining a unique type of transmission, which differs from the clinical disease state.

To avoid confusion, we use the term “transmission” to refer to the intercellular transport of seeds or the cellular incorporation of exogenous seeds, and the term “propagation” to refer to the increase in misfolded a-syn induced by the seeds.

## Materials and methods

### Antibodies

The antibodies used in the study are listed in Additional file 1: Table S1.

#### Preparation of recombinant a-syn

His-tagged a-syn was expressed and purified using and Ni affinity resin. In the last step of the purification protocol, the His tag was eliminated. As a result, the a-syn protein used in this experiment had no tag attached. We removed the tag because it might affect propagation and aggregation. Both, human and mouse a-syn were used in the study. The *Escherichia coli* strain BL21 (DE3) was transformed with the expression vector pET15b, encoding wild type (WT) human or mouse a-syn. The expression of His-tagged a-syn proteins was induced by the addition of 0.5 mM isopropyl β-d-thiogalactoside at 37 °C for 3 h. Cells were lysed by ultrasonication in PBS containing 2% Triton X-100, centrifuged at 20,000×g for 30 min. The supernatant thus obtained was loaded on a Ni Sepharose 6 Fast Flow column (1 mL, GE Healthcare). a-Syn was eluted with a buffer containing 50 mM Tris-HCl, 100 mM NaCl, and 250 mM imidazole, at pH 8.0. The eluted samples were concentrated by centrifugation at 3000×g for 15 min using Vivaspin Turbo (5 K MW) tubes (15 mL) with buffer containing 50 mM Tris-HCl and 100 mM NaCl, pH 8.0. Proteins were treated with thrombin (GE Healthcare) to remove the N-terminal His-tag.

#### Fibril formation

Purified a-syn monomers (100 μM, 150 μl) were incubated at 37 °C in a shaking incubator at 1200 rpm, in 50 mM Tris–HCl containing 100 mM NaCl (pH 8.0), for 5 days. Measurements at OD 600 (or other wavelengths) were used to check turbidity. After 5 days a-syn pre-formed fibrils (PFFs) were pelleted by spinning at 50,000×g for 20 min and suspended in PBS.

#### Animals

C57BL/6J mice were obtained from CLEA Japan, Inc. All breeding, housing, and experimental procedures were performed according to the guidelines for Animal Care of Juntendo University and approved by the Juntendo University Animal Care and Use Committee. Only male mice were used for this study.

#### Seeds injection

We sonicated a-syn PFFs before the intracerebral injection (using Bioruptor UC100-D2, TOS; 20 pulses; each pulse consisting of a 20-s ‘ON’ period and a 20-s ‘OFF’ period). Mice ranging between 2 to 3 months of age were anesthetized using an isoflurane/oxygen/nitrogen mixture and were unilaterally injected with 5 μg/2.5 μl of recombinant mouse or human a-syn PFFs into the right striatum (A-P: 0.2 mm; M-L + 2.3 mm; D-V: − 2.6 mm, from bregma) using a 10 μL Hamilton syringe at a rate of 0.1 μl per min. Control animals received sterile PBS. Mice were anesthetized with an isoflurane/oxygen/nitrogen mixture and killed by decapitation at various pre-determined time points (1 week, 0.75, 1.5, 3, and 6 months). For histological studies, mice were perfused with PBS followed by 4% paraformaldehyde (PFA) in PBS followed by overnight incubation of the tissue post-fixation, in either neutral buffered formalin (Fisher Scientific) or 70% ethanol before undergoing processing and embedding in paraffin.

#### Callosotomy

We used a surgical stitching needle (straight, 17-mm long), the tip of which was filed down with sandpaper. An incision was made from bregma, extending 3 mm anteriorly and 4 mm posteriorly, cutting in a continuous line perpendicular to the cerebral ventricle, with the needle at a depth of 3 mm. All incisions were made 0.4 mm to the left of bregma. The corpus callosum was severed either 1 day before or 1 day after the a-syn PFFs injection, and dissection was performed 1.5 months later.

#### Botulinum toxin B (BoNT/B) injection

BoNT/B was used in this study. NerBloc (rimabotulinumtoxin B) 2500 units/500 μL solution was purchased from Eisai. In total, 10 units/2 μL of BoNT/B was administered to the left striatum of each mouse, according to the stereotaxic surgical procedure described above (A-P: 0.2 mm, M-L: − 2.3 mm, D-V: − 2.6 mm from the bregma). BoNT/B was administered either 3 days prior to or 1 day after a-syn PFFs injection.

#### Tissue preparation

Mice were perfused with PBS, followed by 4% PFA in PBS. To prepare paraffin sections, brains were post-fixed, dehydrated, and embedded in paraffin wax. Sections of 5-μm thickness were cut with an HM430 sliding microtome (Leica).

#### Immunohistochemistry

Autoclaved paraffin sections were incubated with blocking solution containing 5% skim milk in TBST (20 mM Tris-HCl, pH 8.0, 150 mM NaCl, 0.05% Tween 20) for 1 h. Sections were incubated with the primary antibodies in TBST overnight at 4 °C, followed by the secondary antibodies. For diaminobenzidine (DAB) staining, sections were quenched with 3% H_2_O_2_/methanol for 30 min before blocking and incubated with the VECTASTAIN Elite ABC Kit reagent (Vector Laboratories) for 30 min after secondary antibody incubation. Color development ensued using 3,3′-diaminobenzidine/H_2_O_2_. For the immunofluorescent study, sections were incubated with appropriate fluorescent secondary antibodies conjugated with Alexa-Fluor 488 or 594 (Invitrogen). After washing, sections were mounted to coverslips with VECTASHIELD Mounting Medium (Vector Laboratories).

For human samples, formalin-fixed autopsied brains (midbrains) of two separate PD patients were provided by the neuropathologic library of Juntendo Neurology. Sections of 6-μm thickness were cut with an HM430 sliding microtome (Leica).

For human samples, before mounting with VECTASHIELD Mounting Medium, potential lipofuscin autofluorescence in the tissue sections was quenched using the TrueBlack Lipofuscin Autofluorescence Quencher (Biotium).

The Images were taken with a BIOREVO BZ-9000 and BZ-X700 (Keyence), TCS SP5 confocal microscope (Leica), and an inverted laser scanning confocal microscope (Zeiss LSM 880, Carl Zeiss), using a 63× oil immersion objective and the ZEN Software (Carl Zeiss). Care was taken to capture approximately similar regions in each comparative sample. For counting phosphorylated a-syn (p-syn) inclusions, whole-brain sections were imaged with a Keyence microscope (BZ-9000) using bright field capture. Multiple fields were captured using a 10× objective and stitched together using the Keyence Merge function. The p-syn deposits per area were quantified using the BZ-9000 Generation II Analyzer (Keyence) Single Extraction function of the Hybrid Cell Count software, based on hue (details are described in Additional file 1: Figure S11).

#### Statistics

Sample sizes were determined on the basis of pilot experiments and previous data from similar experiments. To examine whether the samples had the same variances, we first analyzed them with an F-test. Data are presented as mean ± SEM. In Fig. 2b-f, 3d-f, 4c-d, the quantitative comparison of p-syn positive inclusions between the a-syn PFFs administered side (ips) and the contralateral side (contra) was analyzed using a paired t-test. In Fig. 2, the mixed effect model with contrast-based test was carried out to evaluate the significance of trends in time related response using the linear contrast. In Fig. 4b, analysis of covariance (ANCOVA) was conducted by adjusting for the area factor (ips, contra) to examine the time related response. For Fig. 5, we used an unpaired Student’s t-test with Bonferroni correction (*p < 0.05, **p < 0.01, ***p < 0.001 and ****p < 0.0001). A *p*-value of < 0.05 was considered statistically significant.

## Results

### a-Syn PFF seeds are transported in both retrograde and anterograde directions

**Fig. 1:**
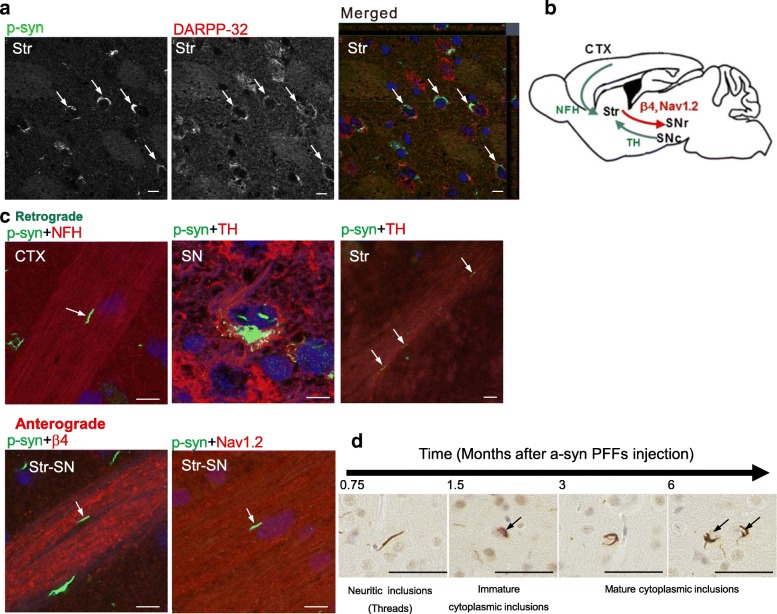
a-Syn inclusions were observed in the axons of the input and output of neural connections in the striatum. An immunohistochemical evaluation of p-syn inclusions in the input and output of neural connections in the striatum (Str) was conducted in the brains of mice 6 months after the injection of mouse a-syn PFFs seeds into the striatum. **a** Medium spiny neurons (MSN) of the striatum stained with anti-DARPP-32 antibody (DARPP-32, red) showed anti-p-syn #64 (p-syn, green)-positive deposits. Z-stack confocal images are shown as merged images. Side views are examined the xz and zy planes. **b** Schematic of the input and output fibers of the striatum. CTX: cortex, Str: striatum, SNpc: substantia nigra pars compacta, SNr: substantia nigra pars reticulata. **c** Double staining of the anti-p-syn#64 (p-syn, green) and each axonal marker for the input and output of the striatum. In the retrograde direction, p-syn deposits (threads) were detected in axons from the cortex, stained with anti-neurofilament H antibodies (NFH, red) (upper panel, left), and in axons from the SNpc, stained with anti-tyrosine hydroxylase antibody (TH: red) (upper panel, center and right). In the anterograde direction, p-syn deposits were detected in axons from the striatum to the SNr, stained with anti-mSCN4B-C antibodies (β4, red) (lower panel, left), or with anti-Nav1.2 antibodies (Nav1.2, red) (lower panel, right). Arrows indicate p-syn in the axons, with each axonal marker. **d** Following injection of a-syn PFFs into the striatum, at 0.75,1.5, 3, and 6 months, the typical shape of the inclusions was evaluated as shown. Scale bars, 10 μm (**a, c**), 50 μm (**d**)

To elucidate whether the a-syn pathology spreads through neural circuits in the brain, recombinant mouse a-syn PFFs (Additional file 1: Figure S1) were injected as “seeds” into the right dorsal striatum of C57BL/6J mice. Immunohistological methods were then used to examine in detail the expansion of a-syn pathology in the mouse brains 6 months later. Cytoplasmic inclusions and neuritic inclusions (threads) were identified as phosphorylated a-syn (p-syn) accumulations using anti-phospho-alpha-synuclein antibody (anti-p-syn #64). These pathological accumulations were observed in the medium spiny neurons (MSNs) of the striatum, identified as DARPP-32-positive neurons (Fig. 1a). In addition, pathological p-syn accumulations were observed in the tyrosine hydroxylase (TH)-positive neurons of the substantia nigra pars compacta (SNpc), which project their axons into the striatum (Fig. 1b-c). Orthogonal projection studies identified an anti-p-syn#64-positive deposit (green) inside the DARPP-32-positive neuron or the TH-positive neuron (red) (Fig. 1a and Additional file 1: Figure S2), suggesting the presence of p-syn uptake in these cells. Thread-like deposits were detected not only in TH-positive axons but also in sodium channel β4 subunit (β4)-positive [30] or sodium channel, voltage-gated, type II (Nav1.2)-positive axons, which project to the globus pallidus or substantia nigra pars reticulata (SNr) from the striatum, as well as in the neurofilament H-positive axons that project to the striatum from the cortex (Fig. 1b-c). Images from the orthogonal projection demonstrate the localization of p-syn in axonal projections. Additionally, the anti-p-syn#64-positive deposits (green) located in apposition to each axonal marker (red). These results strongly suggest that p-syn forms aggregated or was transported in those axons.

Next, to evaluate how the size of a-syn deposits changed over time, we performed a chronological analysis at 0.75, 1.5, 3, and 6 months following injection with mouse a-syn PFFs. The a-syn deposits propagated gradually following injection, and they developed from thread-like or faint cytoplasm deposits, at 0.75 months, to concentrated, perinuclear tangle-like inclusions (hereafter called cytoplasmic inclusions) at 3 to 6 months (Fig. 1d). The formation and distribution of these inclusions following injection with a-syn seeds are displayed chronologically in histograms (Additional file 1: Figure S3). There was a tendency for the aggregates to be larger and more numerous in the a-syn PFFs-injected (ipsilateral) side compared with those in the contralateral side, which consisted of many smaller-sized inclusions, such as threads. However, the largest inclusions on each side of the brain were almost the same size (Additional file 1: Figure S3). It is possible that a-syn deposits gradually increase in size (propagate) at each transmitted (disseminated) site. In contrast to the injection of a-syn seeds, no pathological a-syn inclusions were found at any sites at any point in time, when the injection consisted of phosphate-buffered saline (PBS) alone, as a negative control (Additional file 1: Figure S4).

Double staining with glial cell markers and anti-p-syn#64 were conducted to investigate whether there was an uptake of a-syn seeds by other cell types, in addition to neurons (Additional file 1: Figure S5). The localization of p-syn (green) within iba-1 (red) positive cells was also observed, although this occurred infrequently. This too was confirmed through orthogonal projection. Although infrequent, double staining with antibodies against the oligodendrocyte marker GST-pi and p-syn revealed p-syn deposits in some oligodendrocytes of the white matter. In contrast, anti-GFAP staining showed that astrocytes did not have any p-syn inclusions.

### a-Syn PFF seeds are transmitted through neural connections

**Fig. 2:**
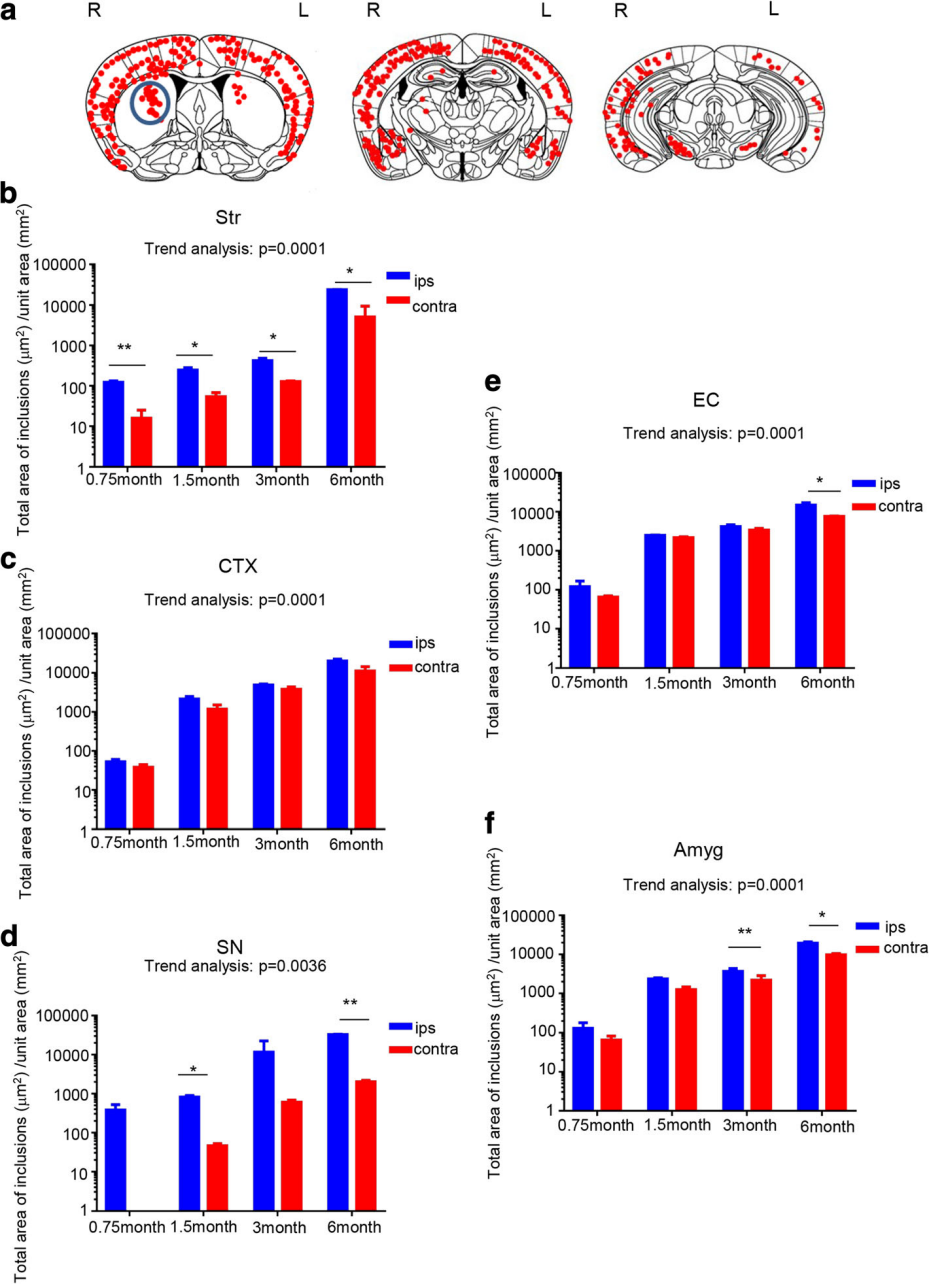
P-syn inclusions were found in neural systems directly or indirectly connected with the striatum where a-syn seeds were injected. **a** Distribution of p-syn pathology in a-syn PFFs-injected mouse brain 6 months after injection. Red dots indicate neuritic inclusions (threads) or cytoplasmic inclusions. Blue circles indicate the injection site in the striatum. L = left hemisphere of the brain; R = right hemisphere. (**b-f**) Total area of the p-syn-positive inclusions (deposits) quantified chronologically at 0.75, 1.5, 3, and 6 months for each region (Str, CTX, SN, EC, and Amyg) of the brain. Horizontal axis: Time following a-syn PFFs injection; vertical axis: Total area of p-syn-positive inclusions (μm^2^) per unit area (mm^2^). **b** Str (0.75 month *p* = 0.0062, 1.5 month *p* = 0.031, 3 month *p* = 0.0413, 6 month *p* = 0.0117), **c** CTX, **d** SN (1.5 month *p* = 0.0122, 6 month *p* = 0.0013), **e** EC (6 month *p* = 0.0235), and **f** Amyg 3 month *p* = 0.0025, 6 month *p* = 0.0189. Mixed effect model with contrast-based testing: Fig. 2b *p* < 0.0001, Fig. 2c *p* < 0.0001, Fig. 2d *p* < 0.0001, Fig. 2e *p* < 0.0001, Fig. 2f *p* < 0.0001. Data is represented as mean area per region ± SEM, *n* = 5 mice per group. Paired *t*-test and the mixed effect model with contrast-based test was carried out to evaluate the significance of trends in time related response using the linear contrast; *p < 0.05, **p < 0.01. Str, striatum; CTX, cortex; SN, substantia nigra; EC, entorhinal cortex; Amyg, amygdala

The distribution of a-syn inclusions at 6 months post-injection is illustrated in Fig. 2a. Accumulation of a-syn was seen in regions with both direct and indirect connections to the injected striatum (Fig. 2a).Time-dependent propagation of pathological a-syn was detected between 0.75 and 6 months (Fig. 2b-f, Additional file 1: Figure S6). The mixed effect model with contrast-based testing was carried out to evaluate the significance of trends in time related to response using the linear contrast. Significant trends in time related to response were observed in striatum, cortex, substantia nigra (SN) and entorhinal cortex (EC), and the amygdala (Amyg). The accumulation of pathological a-syn spread from the injected site to the ipsilateral cortex, contralateral cortex (Fig. 2c), contralateral striatum (Fig. 2b), ipsilateral and contralateral Amyg (Fig. 2f), and ipsilateral SN (Fig. 2d); with the contralateral striatum and contralateral SN demonstrating delayed accumulation. In addition, despite the lack of a direct fiber connection to the striatum, accumulation of pathological a-syn was also detected in the EC (Fig. 2e), which has direct connections to the Amyg and cortex. On the side contralateral to the injection, propagation of a-syn deposits was detected in a time-dependent manner, between 0.75 and 6 months. In the striatum (Fig. 2b), SN (Fig. 2d), and Amyg (Fig. 2f), the total area of pathological a-syn accumulation tended to be small, in the side contralateral to the injection. These regions are connected via several synapses from the injected side of the striatum.

### Callosotomy inhibited propagation of a-syn deposits in the contralateral side

**Fig. 3:**
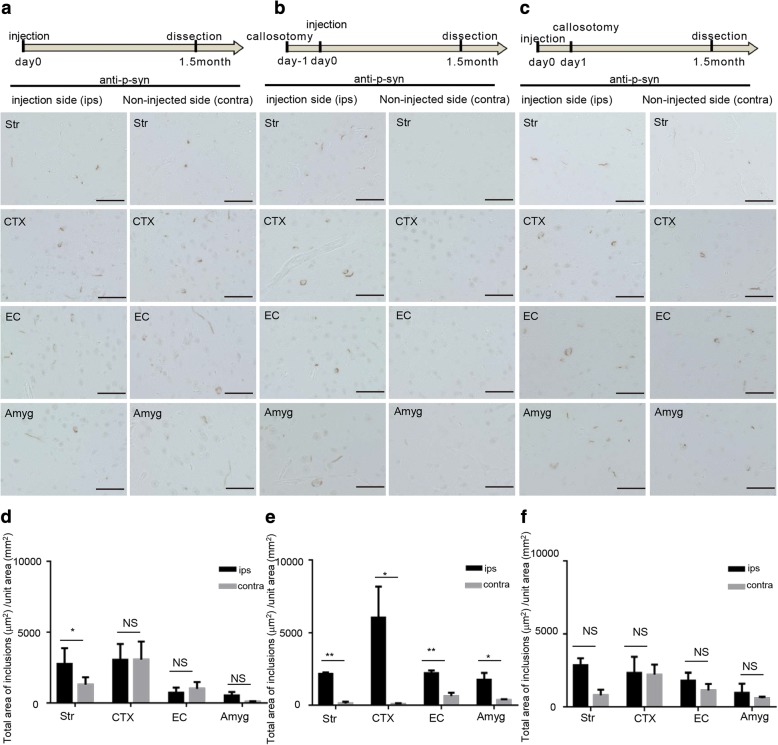
The propagation of a-syn deposits in the contralateral hemisphere was reduced after callosotomy. Callosotomy was conducted 1 day before or 1 day after injection with mouse a-syn PFFs. As a control, a-syn PFFs were injected into mice without callosotomy. (**a-c**)Schematic representation of the experimental protocol and p-syn deposits detected with anti-p-syn#64. Scale bars, 50 μm. (**a, d**) Injection with a-syn PFFs only (no callosotomy) (Str *p* = 0.0171). (**b, e**) Callosotomy 1 day before injection with a-syn PFFs (Str *p* = 0.0098, CTX *p* = 0.0205, EC *p* = 0.0025, Amyg *p* = 0.0476). (**c, f**) Callosotomy 1 day after injection with a-syn PFFs. (**d-f**) The total area of p-syn-positive inclusions (deposits) was quantified for each region (Str, CTX, EC, Amyg) of the brain. Horizontal axis: Brain region; Vertical axis: Total area of p-syn-positive inclusions (μm^2^) per unit area (mm^2^). Data is represented as mean area per region ± SEM, *n* = 5 mice per group, paired *t*-test; *p < 0.05; **p < 0.01. Str: striatum, CTX: cortex, EC: entorhinal cortex, Amyg: amygdala

Further, to examine whether transmission of a-syn seeds occurred through specific neural circuits, we investigated the impact of disrupting neural circuits (by callosotomy) (Additional file 1: Figure S7) on the transmission and propagation of pathological a-syn in the side of the brain contralateral to the injection site. A callosotomy was performed either 1 day before or 1 day after the injection of mouse a-syn PFFs into the right striatum (Fig. 3b-c and e-f). Compared to the mice in which mouse a-syn PFFs were injected without callosotomy (Fig. 3a, d), mice that received callosotomy before the injection showed a clear reduction in the transmission and propagation of pathological a-syn to the contralateral side (Fig. 3b, e). In contrast, in the mice in which callosotomy was performed 1 day following injection of seeds, pathological a-syn propagation was found in both the cortex and striatum of the contralateral side (Fig. 3c, f).

### Extrinsic seeds first moved to the contralateral side within 24 h and then gradually propagated to form aggregates

**Fig. 4:**
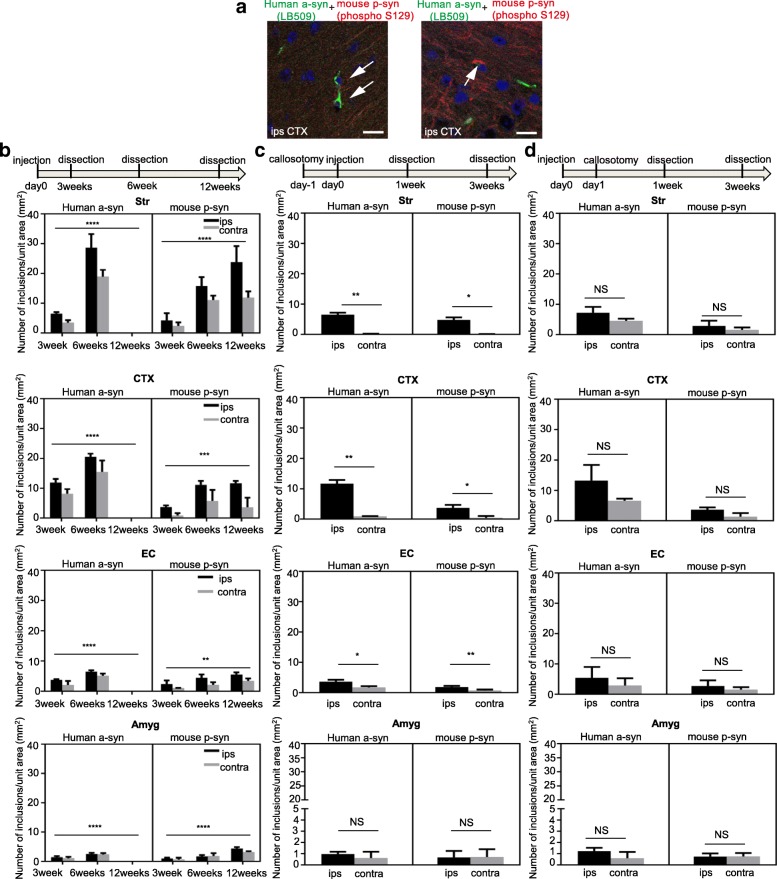
Extrinsic a-syn seeds were transmitted to the contralateral side within 24 h. **a** Mouse brains were stained with human a-syn-specific antibody LB509 (green) and anti-p-syn antibody (phospho S129) (red). Scale bars, 10 μm. **b** The number of human a-syn (left panels) and mouse a-syn (right panels) inclusions after human a-syn PFFs injection was quantified chronologically in each region of the brain: Str (Human a-syn *p* < 0.0001, mouse p-syn *p* < 0.0001), CTX (Human a-syn *p* < 0.0001, mouse p-syn *p* = 0.0008), EC (Human a-syn *p* < 0.0001, mouse p-syn *p* = 0.0014), and Amyg (Human a-syn *p* < 0.0001, mouse p-syn *p* < 0.0001). Data is represented as mean number of a-syn inclusions per region ± SEM, n = 5 mice per group, analysis of covariance (ANCOVA) was conducted to adjust area factor (ips, contra) to examine time related response. **c** Callosotomy 1 day before injection with human a-syn PFFs. **d** Callosotomy 1 day after injection with human a-syn PFFs. (**c, d**) The number of human a-syn (left panels) and mouse p-syn (right panels) inclusions (deposits) was quantified chronologically in each region (Str Human a-syn *p* = 0.0024, mouse p-syn *p* = 0.0114, CTX Human a-syn *p* = 0.0040, mouse p-syn *p* = 0.0484, EC Human a-syn *p* = 0.0216, mouse p-syn *p* = 0.0015, Amyg) of the brain. Horizontal axis: Time after human a-syn PFFs injection; Vertical axis: Number of a-syn inclusions/unit area (mm^2^). Data are the mean number of a-syn inclusions per region ± SEM, *n* = 5 mice per group, paired *t*-test for mouse and human a-syn at 3 weeks in CTX, Str, EC and Amyg for **c** and **d**. *p < 0.05,**p < 0.01,***p < 0.001, ****p < 0.0001. Str: striatum, CTX: cortex, EC: entorhinal cortex, Amyg: amygdala

To detect exogenous a-syn seeds directly and differentiate them from a-syn deposits produced from the endogenous protein, human a-syn PFFs were injected as seeds into the right dorsal striatum and detected with LB509, an antibody specific to human a-syn. LB509 recognizes human a-syn, regardless of the presence or absence of phosphorylation. In contrast, anti-p-syn antibody (phospho S129, Abcam), also used in this experiment, recognizes both mouse and human phosphorylated a-syn. Therefore, p-syn deposits were determined to be mouse-derived when they were recognized by this anti-p-syn antibody (phospho S129) but not by LB509 (Fig. 4a, Additional file 1: Figure S8). Our results indicate that the exogenous human a-syn PFFs were detected only by LB509 at 3 and 6 weeks (0.75 and 1.5 months), at which times no staining with the anti-p-syn antibody was detected (phospho S129) (Fig. 4a). Sections of SN from autopsied brains of patients with PD were double stained with two antibodies, LB509 and phospho S129. Stained Lewy bodies were detected by both LB509 and phospho S129 and were colocalized. (Additional file 1: Figure S8). Mouse-derived pathological p-syn accumulations were observed gradually following the seed injection (Fig. 4a-b). Human a-syn seed accumulations were found in the same areas where inclusions were observed after mouse a-syn PFFs were injected, but these visible human a-syn inclusions disappeared after 12 weeks (Fig. 4b). From 3 weeks after injection, pathological mouse p-syn deposits were detected and increased subsequently in the areas where human a-syn seeds spread (Fig. 4b). ANCOVA was conducted to examine time related response, and significant differences were observed in the number of a-syn inclusions in striatum, cortex, SN, EC, and Amyg (Fig. 4b). Further, we examined the effects of callosotomy on the transmission of human a-syn PFF seeds and the propagation of mouse a-syn deposits. Callosotomy of the mouse brains was conducted 1 day before or 1 day after injection with human a-syn PFFs, and dissection was performed 3 weeks afterward. When the callosotomy was performed prior to injection of human a-syn seeds, significantly less human a-syn deposits were found in the striatum, cortex, and EC on the contralateral side, and the accumulation of mouse pathological p-syn was significantly reduced in these areas, as well (Fig. 4c). In contrast, when the callosotomy was performed after human a-syn seeds were injected, human a-syn seeds were found to have transmitted and formed visible inclusions in the contralateral hemisphere, and the accumulation of mouse pathological p-syn was also detected (Fig .4d).

### Inhibition of synaptic vesicle fusion blocks the transmission of a-syn seeds

Thus far, our results appear to support the hypothesis that a-syn seeds are transmitted via synapses, and that synaptic machinery may be involved in neuron-to-neuron transmission. We used botulinum toxin (BoNT) to determine whether the transmission of a-syn seeds was dependent upon synaptic vesicle fusion. BoNT is a sequence-specific endoprotease with precise specificity for its molecular targets, and there are no known off-target interactions. BoNT degrades unique structural factors in the synapse vesicle docking and fusion complex SNARE, which is necessary for the release of neurotransmitters that catalyze membrane fusion. We specifically used BoNT/B, which degrades and deactivates VAMP-2 [19, 26].

**Fig. 5:**
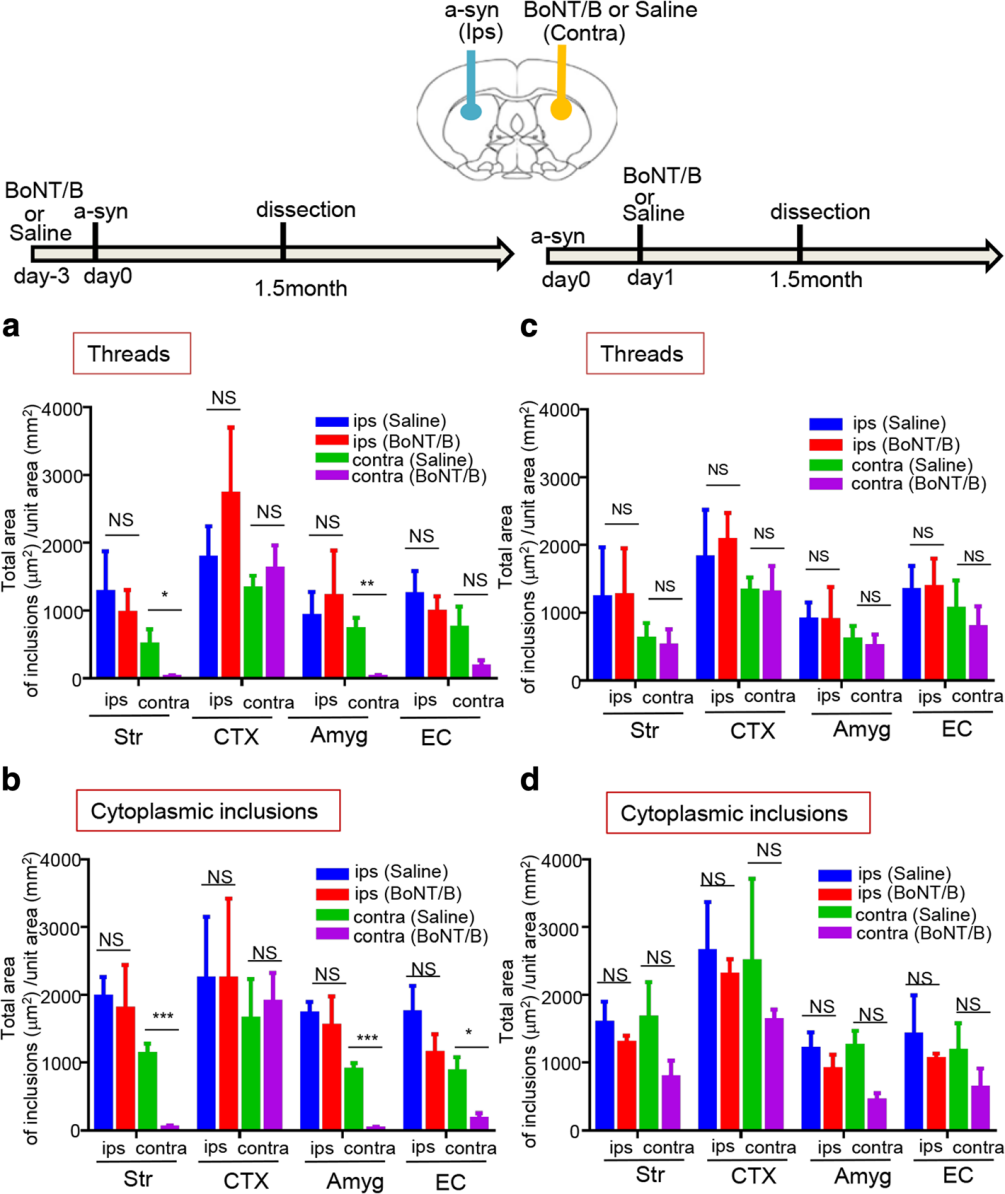
BoNT/B injection into the contralateral side of the seeds injection reduced the propagation of a-syn in the connected areas. Top: Schematic view of the injection sites and the experimental procedures. Injection with BoNT/B to the contralateral side of the seeds injection was conducted 3 days before or 1 day after injection with mouse a-syn PFFs. As a control, physiological saline was administered instead of BoNT/B 3 days before or 1 day after injection of mouse a-syn PFFs. The mice were dissected 1.5 months later. (**a, b**) Injection with saline or BoNT/B 3 days before injection with mouse a-syn PFFs . **a**: Threads (Str *p* = 0.0468, Amyg *p* = 0.0025) **b**: Cytoplasmic inclusions (Str *p* = 0.0002, Amyg *p* = 0.0002, EC *p* = 0.0101). (**c, d**) Injection with saline or BoNT/B 1 day after injection with mouse a-syn PFFs. (**a-d**) The total area of p-syn-positive inclusions (deposits) was quantified for each region (Str, CTX, EC, Amyg) of the brain. Ips: mouse a-syn PFFs injection side, contra: the contralateral side to the mouse a-syn PFFs injection side. Horizontal axis: Brain region; Vertical axis: Total area of p-syn-positive inclusions (μm^2^)/unit area (mm^2^). The p-syn-positive were divided into neuritic inclusions (threads) and cytoplasmic inclusions, and quantified separately. *n* = 5 mice per group, unpaired *t*-test with Student’s correction and Bonferroni correction (significance level 0.05/2 = 0.025); Data are the mean area per region ± SEM; *p < 0.05, **p < 0.01, ***p < 0.001. Str: striatum, CTX: cortex, Amyg: amygdala, EC: entorhinal cortex

BoNT/B was injected into the left (contralateral to the seeds injection site) dorsal striatum, either 3 days prior to, or 1 day after, injection with mouse a-syn PFFs. We evaluated the propagation of p-syn deposits 1.5 months later by immunohistochemistry. Physiological saline was used as a control for the BoNT/B injection. Pathological p-syn deposits in axons (threads) and intracellular p-syn deposits (cytoplasmic inclusions) were quantified separately. When BoNT/B was injected 3 days before the mouse a-syn PFFs injection, we observed that the pathological p-syn accumulation significantly declined in the striatum, Amyg, and EC of the BoNT/B-injected side, compared with control (Fig. 5a,b and Additional file 1: Figure S9). In particular, in the striatum and Amyg, thread-like p-syn deposits decreased (Fig. 5a). A marked reduction in the cytoplasmic inclusions was also observed in striatum and Amyg, as well as EC, which is connected to Amyg (Fig. 5b). In contrast, when BoNT/B was injected 1 day after injection of a-syn seeds, no reduction in a-syn accumulation was observed, similar to what was seen in the control group, injected with physiological saline (Fig. 5c, d). Thus, transmission of a-syn seeds was blocked by BoNT/B, thus our results further confirm that a-syn seeds transmission occurs via synaptic connections.

## Discussion

Accumulating evidence indicates that misfolded protein pathologies can spread throughout the nervous system in a prion-like fashion [2, 17]. It appears that neuronal connections are more important than physical distance for the propagation of a-syn deposits among brain regions. The hypothesis that synaptic transmission of a-syn seeds is responsible for the stepwise expansion of Lewy lesions has been proposed.

In the previous reports, this hypothesis has been discussed from a neuropathological point of view, which stipulated that a-syn accumulation only occurs in the regions where anatomical neural connections exist [4, 17, 19, 25, 28, 29, 33, 40, 41, 49]. Holmqvist et al. have reported that a-syn propagated to the brain stem through the vagus nerve from the intestinal tract [19]. Rey et al. reported that monomers and oligomers of human a-syn injected into the olfactory bulb of mice were transported along the axonal pathways within a few hours [40]. Furthermore, Peelaerts et al. observed that dopaminergic neurons take in all types of a-syn including oligomers, fibrils and ribbons to complete the trans-synaptic transportation [35].In this study, we used callosotomy, a different experimental method from the previous reports, to investigate whether a-syn seeds are specifically transmitted across neural connections, and we experimentally demonstrated the speed of a-syn transmission.

First, in order to confirm the direction of a-syn seeds axonal transport and propagation, we analyzed the accumulation of p-syn in the input and output fibers of the striatum, into which the a-syn seeds were injected. We confirmed that injected a-syn seeds were incorporated into the MSNs of the striatum, to which the a-syn PFFs were administered, and showed the formation of p-syn inclusion bodies. In the neurofilament H-positive axons projecting from the cortex into the striatum, TH-positive axons projecting from neurons in the SNpc, as well as β4- or Nav 1.2-positive fibers projecting from the striatum to the globus pallidus and SNr, thread-like inclusion bodies were detected by anti-p-syn antibody. These results strongly suggest that bidirectional (anterograde and retrograde) transport of seeds occurs in axons from the striatal MSNs, or axons projecting into striatum (Fig. 1a-c).

In our experiments, there were no p-syn inclusions observed inside astrocytes in any of the images. Loria et al. demonstrated that astrocytes degrade a-syn immediately after uptake [23]. Thus it may have been difficult to detect astrocytic p-syn inclusions in the images, obtained at a single time point. In addition, the ability of neurons to degrade a-syn may be lower than that of astrocytes. This presumably indicates that a-syn tends to be accumulated by neurons.

We analyzed the propagation of pathological a-syn over time after the injection of a-syn PFFs into the right striatum. The results showed that accumulation of pathological a-syn tended to increase over time. We observed spreading in the contralateral striatum and in regions connected to the striatum through multiple synapses, such as the contralateral striatum and SN, although the amount of spreading decreased. These data suggest that the transmission of a-syn amounts depended on connectivity. Paumier et al. reported a reduction of the a-syn pathology in SNpc within 180 days after administration of the a-syn fibrils, based on the observation of neuron degeneration [33]. Similarly, Rey et al. observed that the density of the pathology as a whole in the brain tends to decrease over the longer term after administration of the a-syn PFFs [39]. In the present study, brains of mice were analyzed over a period ranging from 0.75 months to 6 months after administration of a-syn PFFs, the p-syn positive deposits in the SN increased within 6 months. The difference could likely be attributed to factors such as the adjustment of synthetic recombinant a-syn. However further longer-term observation is warranted to assess this hypothesis.

Moreover, analysis of the areas and quantities of individual p-syn deposits over time revealed the size of each p-syn deposit to be larger on ipsilateral side as compared to the contralateral side. However, the maximum size of the deposits was equivalent on both sides (Additional file 1: Figure S3). These results suggest that the injected a-syn PFFs initially spread to each area as seeds and formed inclusions over time by recruiting endogenous a-syn. P-syn accumulation in the ipsilateral cortex is the most frequent, and transmission to the striatum and contralateral SN to the site of a-syn PFFs administration tended to be less frequent. This is presumably because the transmission of seeds via multiple synapses is required to reach the striatum and SN on the contralateral side, and the number of seeds declines during transmission.

We further confirmed that a-syn seeds are transmitted through neural circuits using callosotomy (Fig. 3). We designed an experiment using callosotomy to determine whether nerve fiber disconnection inhibits a-syn propagation, in a neural circuit-dependent manner. When callosotomy disconnected the contralateral side from the injected side before the injection of a-syn seeds, the transmission and propagation of pathological a-syn to the contralateral side substantially decreased. This is likely because the delivery of the a-syn seeds to the contralateral side was blocked by severing the axons of the corpus callosum. From this result, we confirmed that the seeds were transported along the path of the nerves. Some propagation of p-syn deposits in the limbic system (EC and Amyg), including routes other than the corpus callosum, such as hippocampal traffic and the anterior commissure, also seem likely to be involved [8], however, the decreased p-syn deposits in these regions after callosotomy suggest that the transmission through the striatum may affect the results through the striatum-Amyg and Amyg-EC connection. In contrast, when the callosotomy was performed 24 h after injection of the a-syn seeds, p-syn accumulated in the contralateral side in a similar fashion as in the control (without callosotomy). This result suggests that exogenous seed migration occurs within a 24-h period.

We also examined the dynamics of the seeds transmission itself. Human a-syn PFFs can be differentiated from mouse a-syn PFFs by the human a-syn-specific antibody LB509 (Fig. 4a). Exogenous human a-syn PFFs injected into the right striatum spread to the contralateral side, in the cortex, striatum, Amyg, and EC, forming visible aggregates (inclusions) 3 weeks (0.75 months) after injection. However, 12 weeks after seed administration, the exogenous human a-syn deposits were no longer detected. Meanwhile, endogenous mouse a-syn inclusions began to appear (Fig. 4b). These results suggest that exogenous human seeds interact with each other and bind other human seeds more rapidly than they convert endogenous mouse a-syn to the misfolded form, due to conformational and species-specific sequence differences (Additional file 1: Figure S10).

The administration of human a-syn PFFs resulted in a slower and reduced formation of a-syn inclusions compared with the administration of mouse a-syn PFFs. This observation has been previously reported [41]. As suggested in previous studies, the species barrier may be the reason for this effect [6]. This observation was also noted by Rey et al. [41]. Further, Luk et al. proposed that the seeding efficiency on a molecular level is determined by the sequence homology of the a-syn PFF seeds and the soluble a-syn monomer, and the result of the present study which showed delayed propagation ability when human a-syn PFFs was administered to mice is also consistent with this proposal [24]. In the present study, the human a-syn deposit itself was undetectable 3 months after seeds administration. We suppose that the majority of exogenous seeds were degraded when the human a-syn deposits disappeared. The later disappearance of human a-syn deposits was also confirmed by the previous studies [2, 3, 24, 29, 39, 41]. Rey et al. stated [39] that this elimination could be due to the degeneration of cells itself, or degradation caused by the autophagy/lysosomal, ubiquitin-proteasome systems [48], or phagocytosis by microglia [7] or astrocytes [23].

Interestingly, the exogenous seeds were not phosphorylated, while the endogenous a-syn aggregates were phosphorylated, suggesting that the exogenous seeds are not phosphorylated inside the cell and can recruit and convert endogenous p-syn more easily than the non-phosphorylated form. Sections of SN from autopsied brains of patients with Parkinson’s disease were double stained with two antibodies, LB509 and phospho S129. Stained Lewy bodies were detected by both LB509 and phospho S129 and were colocalized. Thus, it was inferred that these antibodies did not compete for their epitopes, and this confirmed that the human a-syn PFFs was not phosphorylated. We also examined the spread of the seeds themselves and their speed using callosotomy with the administration of human a-syn PFFs (Fig. 4c, d). When the callosotomy was performed prior to the administration of human a-syn PFFs, transmission to the contralateral side decreased and the emergence of mouse p-syn deposits also decreased. When the callosotomy was conducted 1 day after the injection of human a-syn PFFs, transmission of human a-syn to the contralateral side was observed, and the emergence of mouse p-syn was also observed. From these results, we confirmed that the spread of the exogenous seeds occurs within 24 h. This rapid dissemination/transmission of a-syn phenomenon is very surprising and raises several questions. In clinical cases of PD and dementia with Lewy bodies (DLB)/PD with dementia (PDD), a-syn pathologies spread slowly over a period of 5–10 years [4, 5, 18]. Incidentally, in reports of Lewy body pathology in fetal SN transplants, which provide important evidence for a-syn transmission, it is estimated that Lewy bodies cannot be positively detected until more than 10 years after transplantation [21, 22]. There are several possible explanations for the difference in progression from around 24 h to over 10 years, between experimental transmission systems and the clinic. First, it is plausible that administering artificial protein aggregates to the experimental transmission systems creates a unique situation which does not necessarily reflect the clinical disease. Alternatively, it could be that the seeds themselves, which form the initial explosive trigger for the transmission and aggregation of the pathological protein, can spread within 24 h, while it takes more time for the exogenous seeds to recruit endogenous proteins by altering their conformation. This could represent a novel pathological concept in the disease progression of synucleinopathies. Indeed, since the transmission experiments involve injecting a large dose of seed proteins directly into the brain, they could be viewed as a way to capture the clinical pathological condition in a shorter period of time. Meanwhile, prion disease has been shown to spread extremely quickly after onset, both experimentally and clinically [27]. This difference could arise from the fact that prion disease causes changes in the cell membrane [36] which lead to a rapid worsening of disease, while synuclein causes changes within the cytoplasm [20, 45, 47] which may lead to slower progression.

**Fig. 6:**
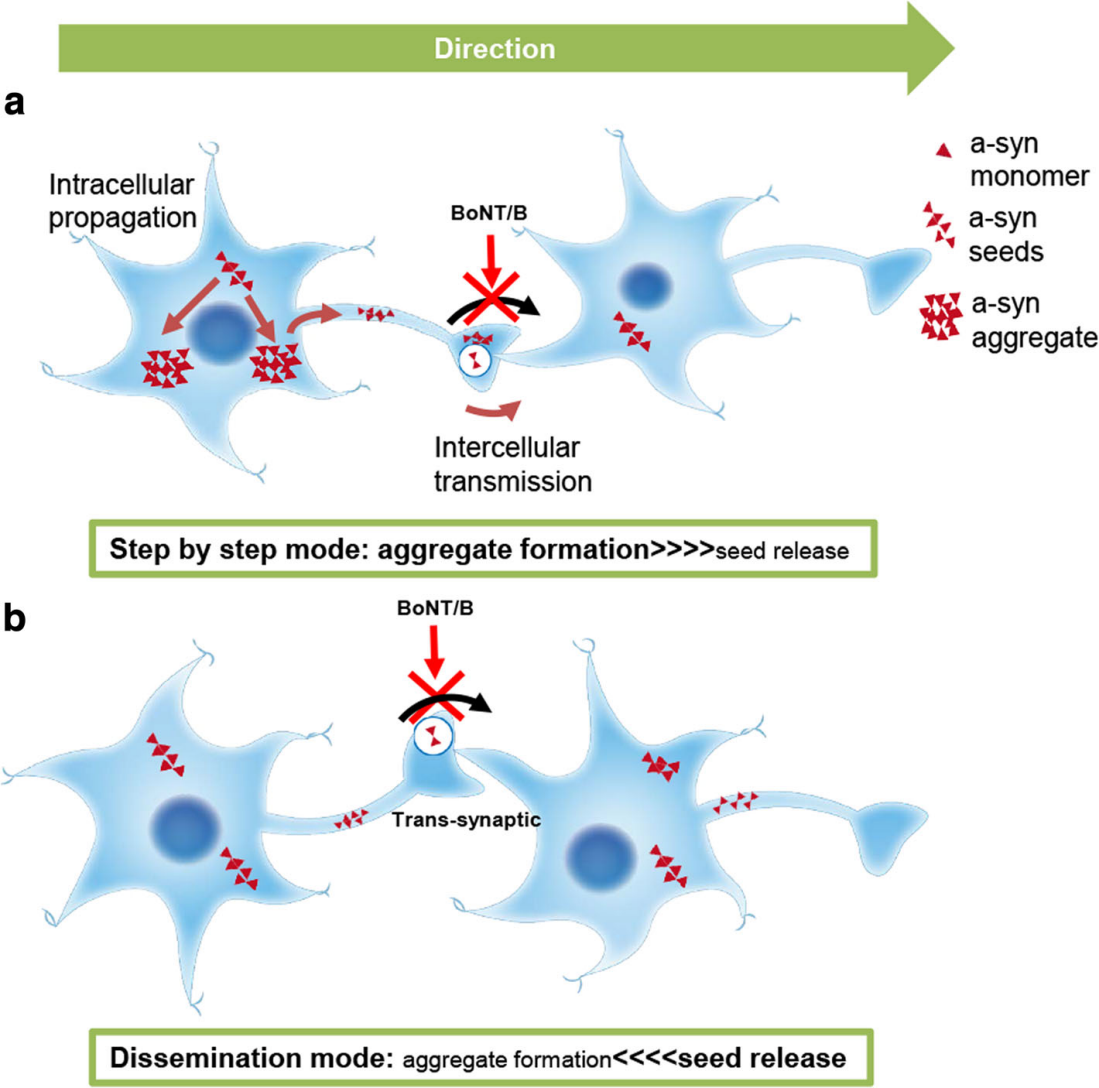
Different modes of a-syn transmission and propagation. **a** Schematic representation of the step-by-step mode, whereby misfolded a-syn propagation forms aggregates, and then seeds are released in a neuron, and finally the seeds are transmitted to a connected neuron via the synapse. **b** Schematic representation for the dissemination mode, whereby seeds are disseminated transneuronally and induce the propagation of misfolded a-syn to form aggregates in the disseminated areas. a-Syn seeds are transmitted trans-synaptically, indicating inhibition due to injection of BoNT/B

In recent years, the propagation of pathological a-syn to anatomically adjacent neurons or cells has been observed in other studies as well, further supporting the trans-neuronal and trans-synaptic transportation of a-syn seeds [4, 9, 19, 28, 33, 35, 40, 41]. However, a study using cultured primary neurons reported that the transmission of pathological a-syn may not necessarily occur via synapses. Indeed, the transmission between axons and soma has been observed during the early culture phase (1 to 4 days), prior to the formation of synapses [11]. There are additional studies that report mechanisms other than synaptic transmission, such as transport via nanotubes [1], exocytosis or exosomes, or by receptor-mediated endocytosis [10, 17, 38]. However, our results strongly suggest that a synaptic mechanism underlies the trans-neuronal transmission of a-syn seeds in-vivo. Understanding the pathways involved in the pathological propagation of proteins is an important step for developing therapeutic interventions. The results of the present study using callosotomy suggest that the rapid dissemination of seeds through synapses does indeed occur. Finally, we used BoNT/B to investigate whether the transmission of seeds was inhibited by the cessation of synaptic release (Fig. 5). BoNT blocks synaptic vesicle fusion in the presynaptic terminal by breaking down and inactivating SNARE proteins in a highly specific manner (VAMP-2 by BoNT/B). BoNT has been shown to block vesicle exocytosis, but not endocytosis [31]. The role of VAMP-2 in the presynaptic SNARE complex that mediates vesicle fusion is widely accepted [46]. In our present study, BoNT/B injected into the striatum inhibited the exocytosis of a-syn seeds from the synapses of axons entering the striatum from the cortex. Transmission and propagation of pathological a-syn in the contralateral striatum and Amyg were inhibited. As a result, we observed that BoNT/B treatment reduced accumulation of p-syn in an area in which the seeds transmission should occur, confirming the presence of trans-synaptic transmission using an in vivo model, for the first time. Previously, it was reported that BoNT inhibited the spread of huntingtin protein in primary cultures, but the actual substances used were β-bungarotoxin [34], at least as far as the product numbers suggest. Thus, ours is the first study to definitively demonstrate trans-synaptic transmission of a pathological protein using BoNT in vivo. In recent years, the association of inflammation with PD or other diseases has been discussed. A study reported that inflammation alters neuronal functions, leading to an increase in cell death [43] and another study reported that inflammatory environments enhance a-syn spreading [13]. The possibility that inflammation was caused by a surgical procedure such as callosotomy and injection of BoNT cannot be denied. Thus in this study, we performed callosotomy or injection of BoNT before or after administration of a-syn PFFs. When we performed callosotomy or injection of BoNT before a-syn PFFs administration, the spreading of a-syn inclusions decreased. However, when the administration of a-syn PFFs was performed after callosotomy or injection of BoNT, spreading of a-syn inclusions occurred equally as that with a-syn PFFs administration alone. Therefore, we concluded that inflammation had little effect in this study. Combined with the results of the experiments using callosotomy, we confirmed the rapid transmission and dissemination of a-syn seeds through circuit and synapses. Our findings provide empirical support for a dissemination mode (Fig. 6b) of a-syn propagation, where a-syn seeds are disseminated and then induced to form aggregates, and not a step-by-step mode (Fig. 6a) where each neuron forms inclusions and releases seeds. Typical PD shows only limited affected regions and usually has a slowly progressive course. However, the distribution and rapid dissemination of a-syn seeds observed in this study seems to represent a different point in the recognition of PD, beyond its usual disease course. The dissemination mode may be a mechanism underlying the pathology of dementia with Lewy body disease, in which larger regions are affected in the cortex, including in the rapidly progressive phenotype [12]. Further studies will be necessary to elucidate this regulatory mechanism and to develop therapeutic strategies for synucleinopathies.

## Conclusion

In the current study, even when a callosotomy was performed 24 h after seeds injection, a-syn was observed to have propagated to the contralateral hemisphere of the brain, indicating that seeds dissemination throughout the brain occurs very rapidly, within 24 h of injection. This is a new concept that differs from traditional notions of pathological progression; that is, aggregates are gradually formed from preceding, widely transmitted seeds. Moreover, the synaptic transmission of seeds was confirmed using BoNT in vivo.

## Additional file

Additional file 1: Rapid dissemination of alpha-synuclein seeds through neural circuits in an in-vivo prion-like seeding experiment. (PDF 1420 kb)

## Abbreviations

Amyg: Amygdala
ANCOVA: Analysis of covariance
a-syn: Alpha-synuclein
BoNT: Botulinum toxin
BoNT/B: Botulinum toxin B
CTX: Cortex
DAB: Diaminobenzidine
DLB: PD and dementia with Lewy bodies
EC: Entorhinal cortex
LBs: Lewy bodies
MSNs: Medium spiny neurons
Nav1.2: Sodium channel, voltage-gated, type II
PBS: Phosphate-buffered saline
PD: Parkinson’s disease
PDD: PD with dementia
PFFs: Pre-formed fibrils
p-syn: Phosphorylated a-syn
SN: Substantia nigra
SNpc: Substantia nigra pars compacta
SNr: Substantia nigra pars reticulata
Str: Striatum
TH: Tyrosine hydroxylase
WT: Wild type
β4: Sodium channel β4 subunit
