# Supplementary material for: Rapid dissemination of alpha-synuclein seeds through neural circuits in an in-vivo prion-like seeding experiment
Source: Acta Neuropathol Commun. 2018 Sep 19;6:96. doi: 10.1186/s40478-018-0587-0 (PMC6145187; doi:10.1186/s40478-018-0587-0)
Supplement: Supplementary file 1 — Rapid dissemination of alpha-synuclein seeds through neural circuits in an in-vivo prion-like seeding experiment. (PDF 1420 kb) [file 40478_2018_587_MOESM1_ESM.pdf]

Supplementary informations

Rapid dissemination of alpha-synuclein seeds through neural circuits in an *in-vivo* prion-like seeding experiment

Ayami Okuzumi, Masaru Kurosawa, Taku Hatano, Masashi Takanashi, Shuuko Nojiri, Tomoyuki Yamanaka, Haruko Miyazaki, Saki Yoshinaga, Yoshiaki Furukawa, Tomomi Shimogori, Takeshi Fukuhara, Nobutaka Hattori* and Nobuyuki Nukina*

Journal Name: Acta Neuropathologica Communications

*To whom correspondence should be addressed:

Nobuyuki Nukina

Laboratory of Structural Neuropathology, Doshisha University Graduate School of Brain Science, 1-3 Miyakodanitatara, Kyotanabe-shi, Kyoto 610-0394, Japan

e-mail:nnukina@mail.doshisha.ac.jp

tel&Fax:+81-774-65-7211

Nobutaka Hattori

Department of Neurology, Juntendo University Graduate School of Medicine,

2-1-1 Hongo, Bunkyo-ku, Tokyo 113-8421, Japan

e-mail: nhattori@juntendo.ac.jp

Table S1. List of antibodies

Primary antibodies

Product name	Catalaog No.	Company or Reference	Type	Dilution
#64 (phosphorylated alpha-synuclein (pS129))	015-25191	Wako	mouse monoclonal	1/300
alpha-synuclein (phospho S129)	ab59264	Abcam	rabbit polyclonal	1/400
mouse alpha-synuclein	#4179	Cell Signaling	rabbit polyclonal	1/400
LB509 (human alpha-synuclein specific)	18-0215	Invitrogen	mouse monoclonal	1/400
Tyrosine Hydroxylase (TH)	ab76422	Abcam	chicken polyclonal	1/500
Glial fibrillary acidic protein (GFAP)	Z0334	DAKO	rabbit polyclonal	1/1000
Neurofilament H	AB5539	chemicon	chicken polyclonal	1/1000
iba1	019-19741	Wako	rabbit polyclonal	1/1000
DARRP32	AB1656	chemicon	rabbit polyclonal	1/500
mSCN4B-c		Ref. 22	rabbit polyclonal	1/700
Nav1.2 (Na+CP type IIα (G-20))	asc002	Alomone labs	rabbit polyclonal	1/500
GST-pi	312	MBL	rabbit polyclonal	1/500

Secondary antibodies

Product name	Catalaog No.	Company or Reference	Type	Dilution
Biotinylated anti-rabbit IgG	BA-1000	Vector	goat	1/300
Biotinylated anti-mouse IgG	BA-9200	Vector	goat	1/300
Alexa Flour 488 anti rabbit IgG	A-11034	Life technologies	goat	1/300
Alexa Flour 488 anti mouse IgG	A-11029	Life technologies	goat	1/300
Alexa Flour 546 anti rabbit IgG	A-11035	Life technologies	goat	1/300
Alexa Flour 546 anti chicken IgG	A-11040	Life technologies	goat	1/300
Alexa Flour 546 anti mouse IgG	A-11030	Life technologies	goat	1/300

Supplementary Figure S1.

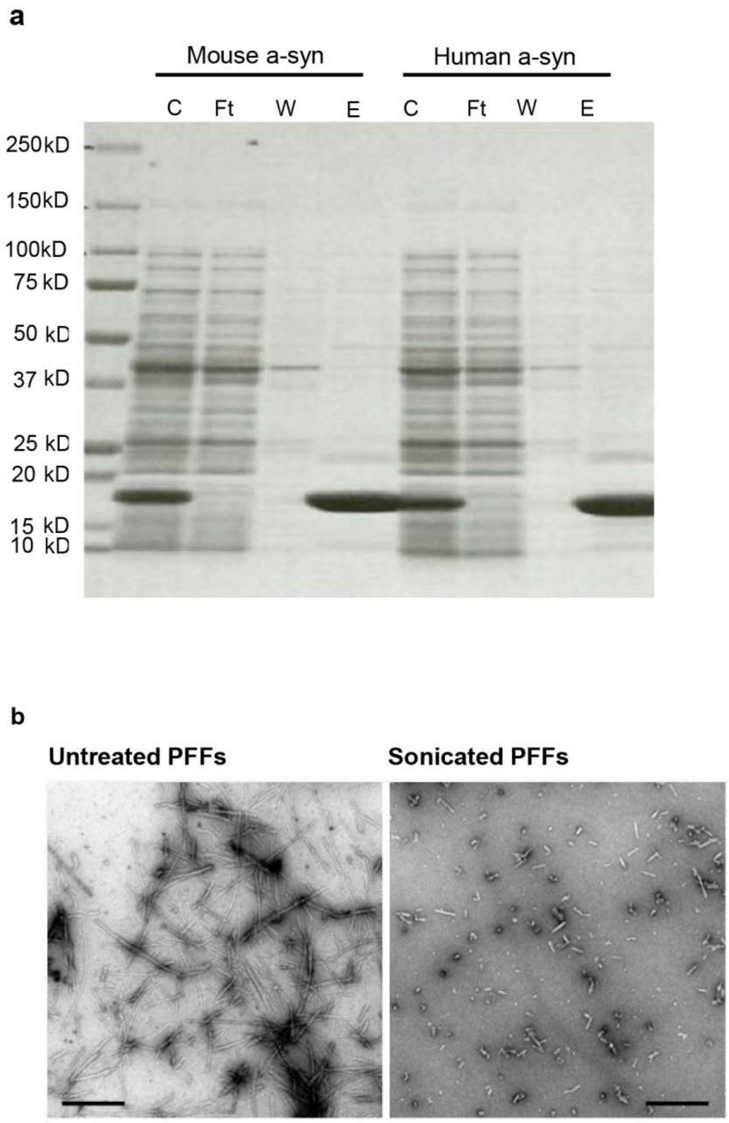

Supplementary Figure S1. Preparation of PFFs

(**a**) His-tagged fusion proteins were purified using Ni Sepharose, and the purification was confirmed by SDS-PAGE using Coomassie Brilliant Blue staining. C: Escherichia coli lysate; Ft: flow-through; W: washing; E: elution fraction. (**b**) Electron microscopy of recombinant a-syn PFFs before and after sonication. Scale bars, 500nm. a-syn: alpha-synuclein, PFFs: preformed fibrils

Supplementary Figure S2.

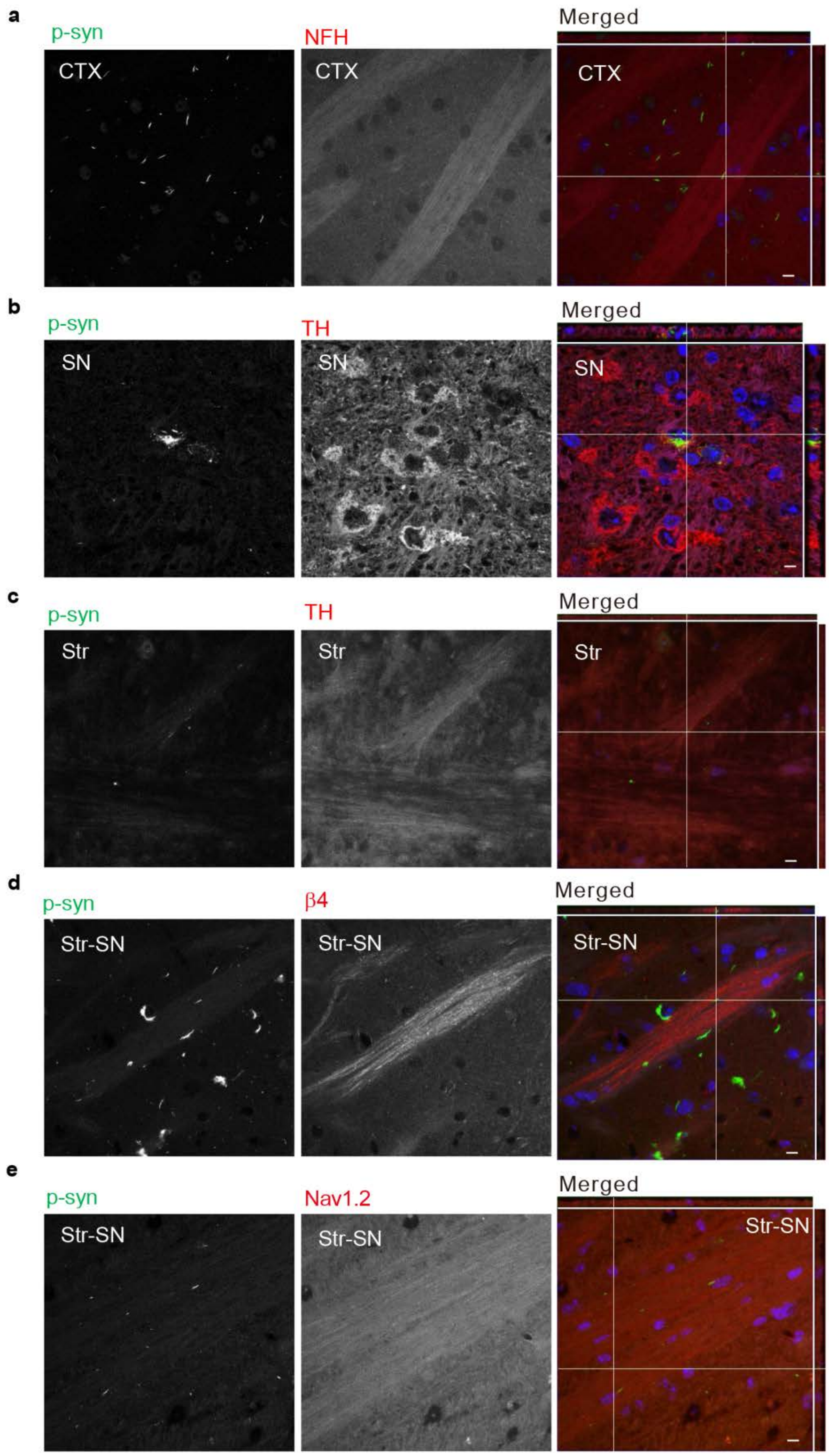

Supplementary Figure S2. Detailed analysis of the localization of p-syn deposits and cell or axon markers was performed using orthogonal projection

An immunohistochemical evaluation of p-syn inclusions in the input and output of neural connections in the Str was conducted in the brains of mice 6 months after the injection of mouse a-syn PFFs seeds into the striatum. Z-stack confocal images are shown as merged images. Double staining of the anti-p-syn #64 (p-syn, green) and each axonal marker for the input and output of the Str. In the retrograde direction, p-syn deposits (threads) were detected in axons from the cortex, stained with anti-neurofilament H antibodies (NFH, red) (**a**), and in axons from the SNpc, stained with anti-tyrosine hydroxylase antibody (TH: red) (**b,c**). In the anterograde direction, p-syn deposits were detected in axons from the striatum to the SNr, stained with anti-mSCN4B-C antibodies (β4, red) (**d**), or with anti-Nav1.2 antibodies (Nav1.2, red) (**e**). Scale bars, 10 μm

Str: striatum, SNpc: Substantia nigra pars compacta, SNr: Substantia nigra pars reticulate, a-syn: alpha-synuclein, PFFs: preformed fibrils, p-syn: phosphorylated a-syn

Supplementary Figure S3.

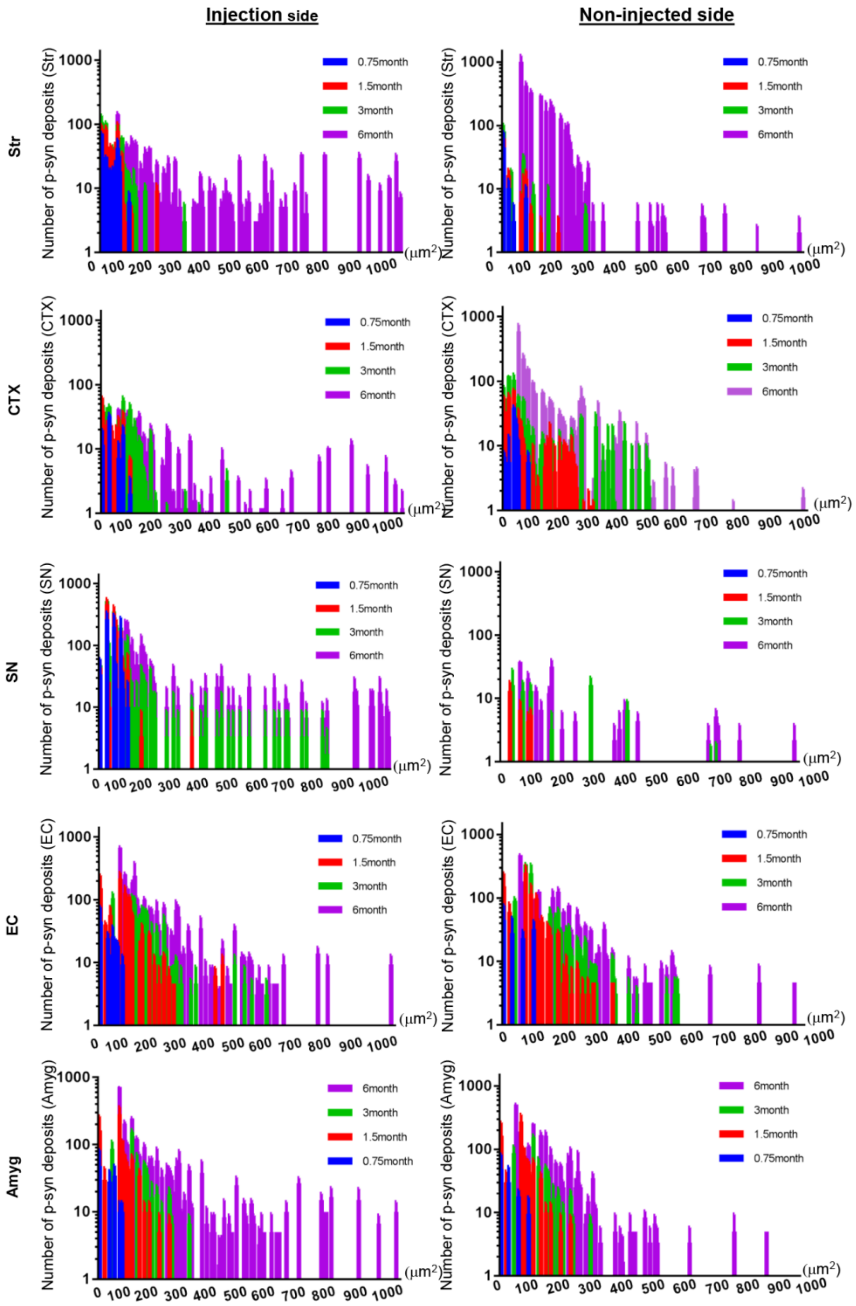

Supplementary Figure S3. a-Syn deposits gradually increased over time in size at each transmitted brain regions

The histograms of the size and number distribution of each a-syn deposit for the different regions. Left: a-syn PFFs injection side; Right: contralateral side to the

a-syn PFFs injection. Horizontal axis: Area of each p-syn deposit; Vertical axis: Number of p-syn deposits per unit area. n = 5 for each duration (0.75, 1.5,3,6 month). Str: striatum, CTX: cortex, SN: substantia nigra, EC: entorhinal cortex, Amyg: amygdala, a-syn: alpha-synuclein, PFFs: preformed fibrils, p-syn: phosphorylated a-syn

Supplementary Figure S4.

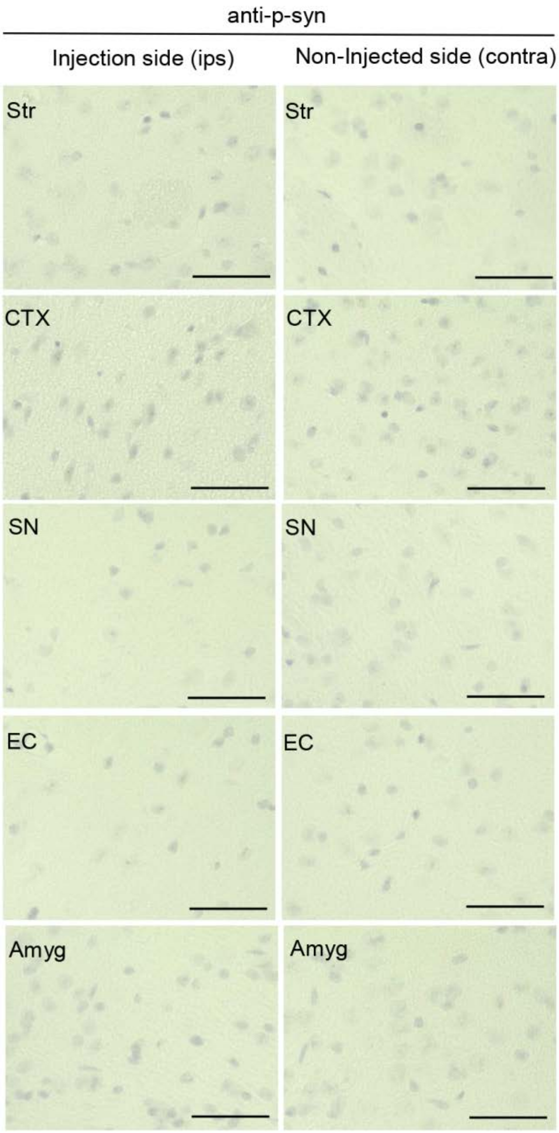

Supplementary Figure S4. PBS injections did not result in pathological a-syn accumulations at any brain regions

Lack of pathological a-syn inclusions at any brain regirons (Str, CTX, SN, EC, Amyg) in mice 6 months after injection with PBS. Immunostaining was conducted using anti-p-syn #64 antibody. Scale bars, 50 μm. Str: striatum, CTX:

cortex, SN: substantia nigra, EC: entorhinal cortex, Amyg: amygdala, a-syn: alpha-synuclein, p-syn: phosphorylated a-syn

Supplementary Figure S5.

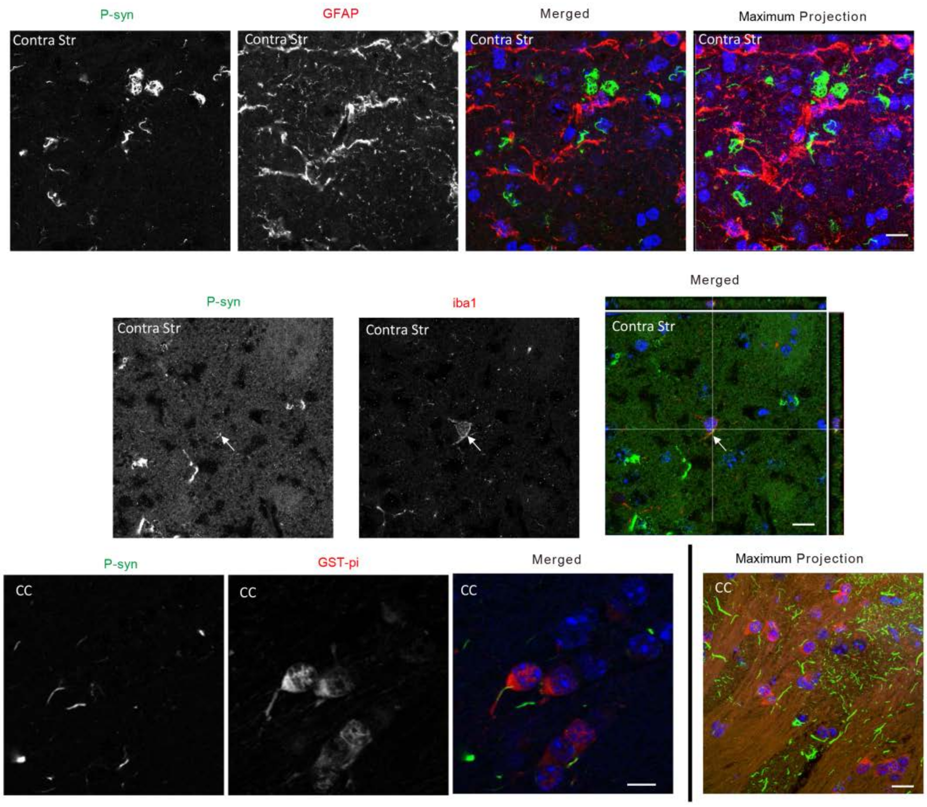

Supplementary Figure S5. P-syn deposits in glial cells

Double stainings with anti-p-syn #64 (green) and each glial cell marker were performed for mouse brains at 6 months after injection of mouse a-syn PFFs into the Str. Anti-p-syn #64 (green) staining with the anti-GFAP antibody (Upper, red; astrocyte marker, Maximum intensity projection shown in right) or the anti-iba1 antibody (middle, red; microglia marker, Z-stack confocal images are shown as

merged images. Side views are examined the xz and zy planes.) in the contralateral Str. Anti-p-syn #64 (green) staining with the anti-GST-pi antibody (Lower, red; oligodendrocyte marker, Maximum intensity projection shown in right side) in the CC. Str: striatum, CC: corpus callosum, p-syn: phosphorylated alpha-synuclein, PFFs: preformed fibrils. Scale bars, 10 μm.

Supplementary Figure S6.

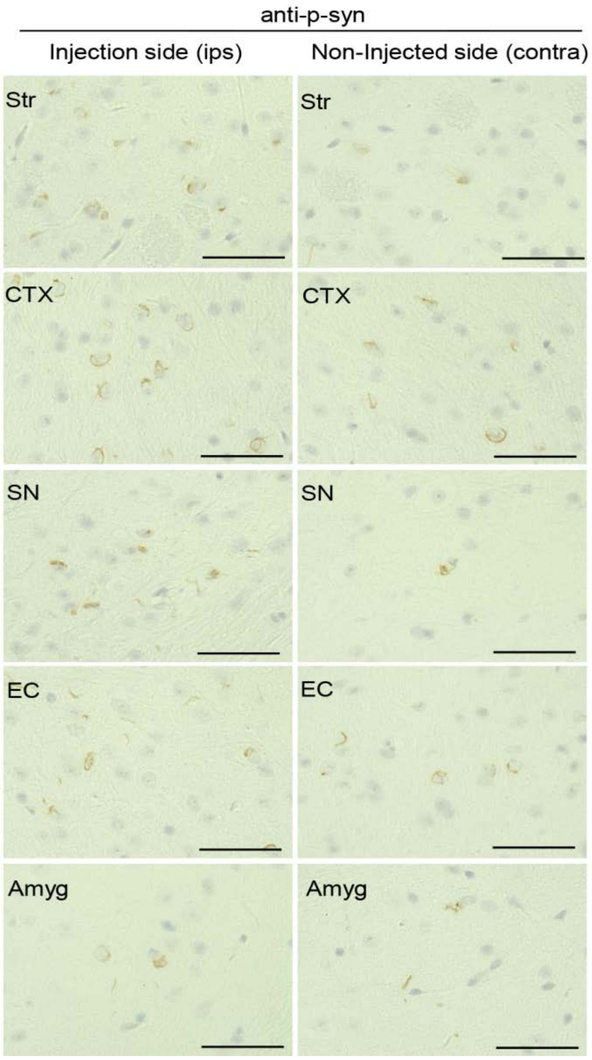

Supplementary Figure S6. P-syn deposits were detected in the different areas at 6 months later after a-syn PFFs injection

Immunostaining was conducted on each region of the brain (Str, CTX, SN, EC and Amyg) using anti-p-syn #64 antibody. Scale bars, 50 μm. Str: striatum, CTX: cortex, SN: substantia nigra, EC: entorhinal cortex, Amyg: amygdala, a-syn: alpha-synuclein, PFFs: preformed fibrils, p-syn: phosphorylated a-syn

Supplementary Figure S7.

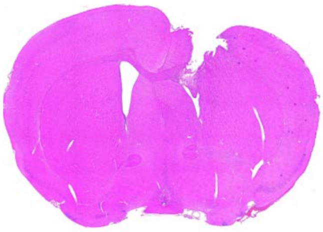

Supplementary Figure S7. An illustration of the coronal section at which callosotomy was performed

This section was stained using Hematoxylin & eosin.

Supplementary Figure S8.

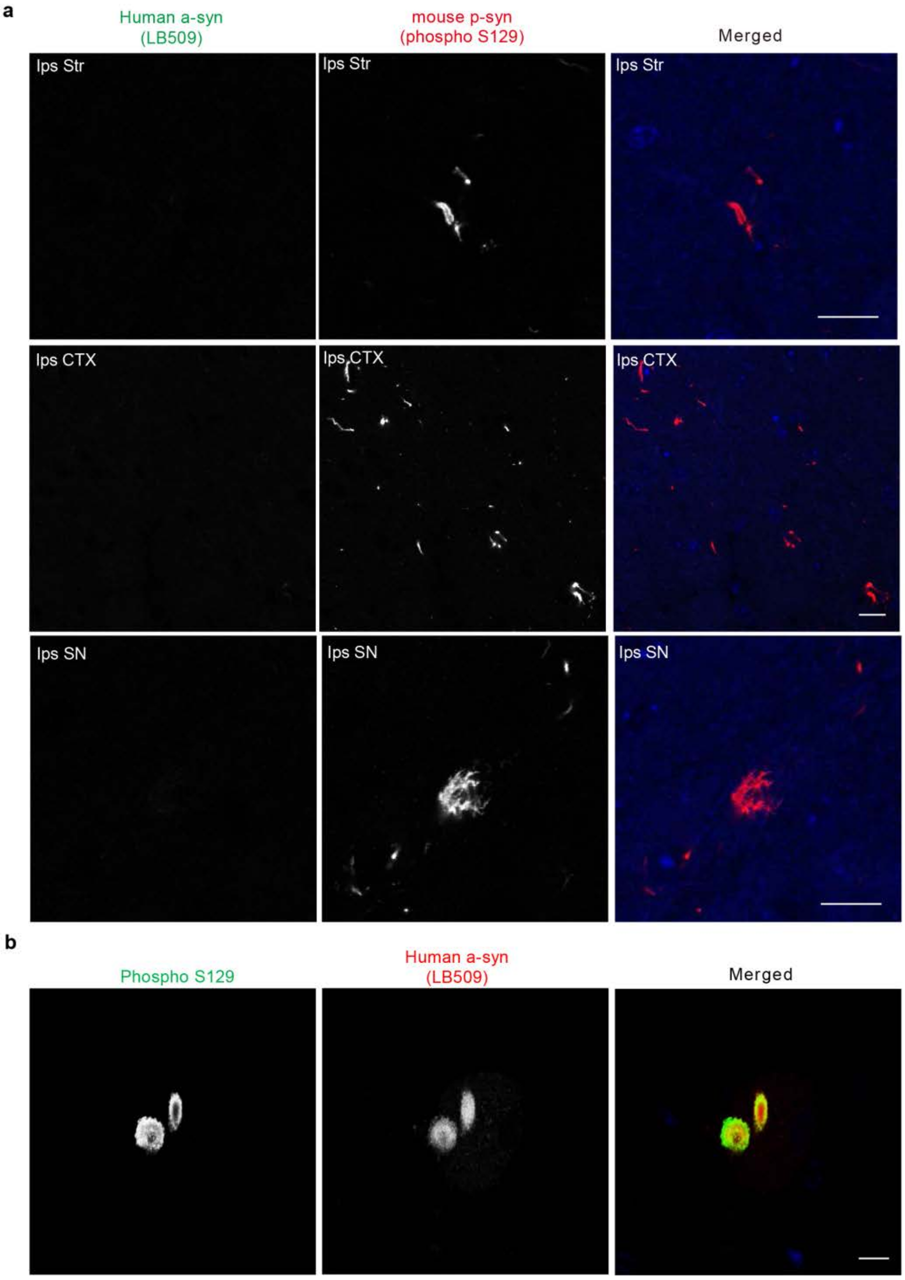

Supplementary Figure S8. Human a-syn-specific antibody LB509 and anti-p-syn antibody (phosphor S129) did not compete for their epitopes

(**a**) Mouse-derived pathological p-syn deposits were recognized by anti-p-syn

antibody (phospho S129) but not LB509. Double stainings with human a-syn-specific antibody LB509 (green) and anti-p-syn antibody (phosphor S129) (red) were performed for each brain regions (Str, CTX and SN) at 6 months after injection of mouse a-syn PFFs into the Str. (**b**) Double staining of Lewy bodies from brains of PD patients using LB509 and anti-p-syn antibody (phospho S129). Formalin-fixed autopsied brains (midbrains) of two separate PD patients were stained using human a-syn-specific antibody LB509 (red) and anti-p-syn antibody (phosphor S129) (green). Lewy bodies were recognized by both anti-p-syn antibody (phospho S129) (green) and LB509 (red). Scale bars, 10 μm. Str: striatum, CTX: cortex, SN: substantia nigra, a-syn: alpha-synuclein, PFFs: preformed fibrils, p-syn: phosphorylated a-syn

Supplementary Figure S9.

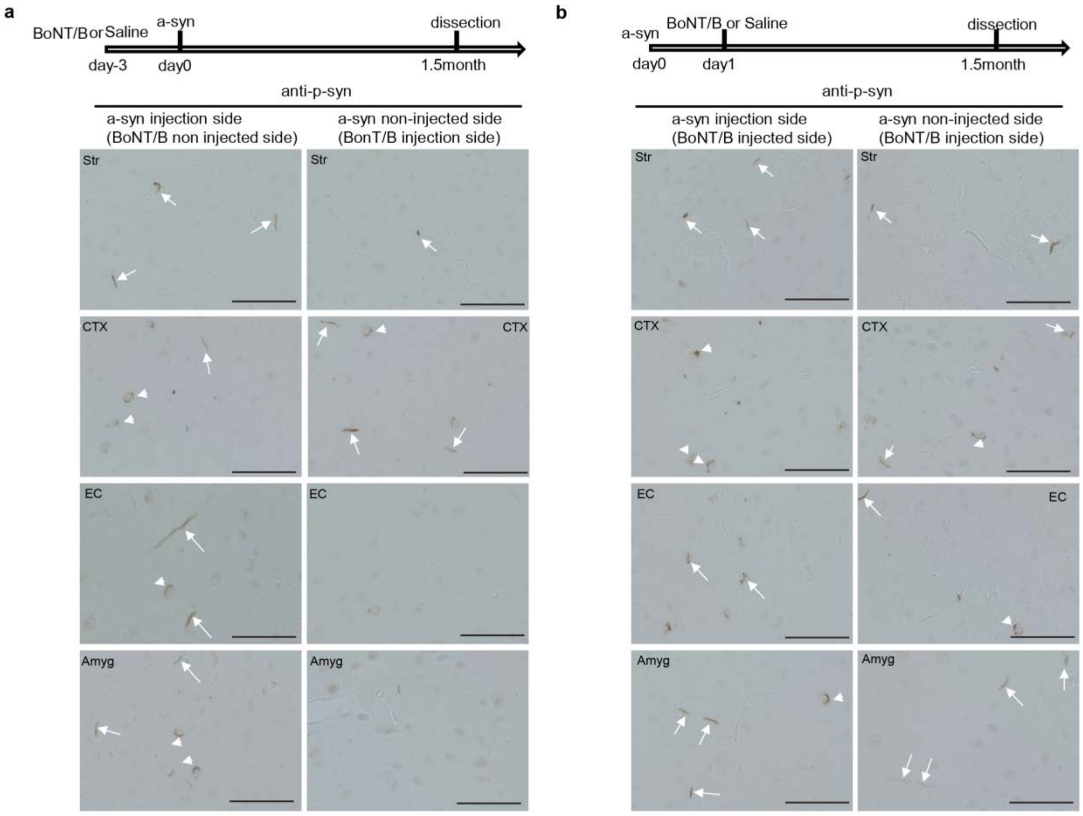

Supplementary Figure S9. BoNT/B injection into the contralateral side of the seeds injection reduced the propagation of a-syn in the connected areas

(**a**) Injection with BoNT/B 3 days before injection with mouse a-syn PFFs. (**b**) Injection with BoNT/B 1 day after injection with mouse a-syn PFFs. (**a, b**) Immunostaining was conducted on each region of the brain (Str, CTX, EC and Amyg) using anti-p-syn #64 antibody. Arrows indicate threads, and arrowheads

indicate cytoplasmic inclusions. Scale bars, 50 μm. BoNT/B: botulinum toxin B, Str: striatum, CTX: cortex, EC: entorhinal cortex, Amyg: amygdala, a-syn: alpha-synuclein, PFFs: preformed fibrils, p-syn: phosphorylated a-syn

Supplementary Figure S10.

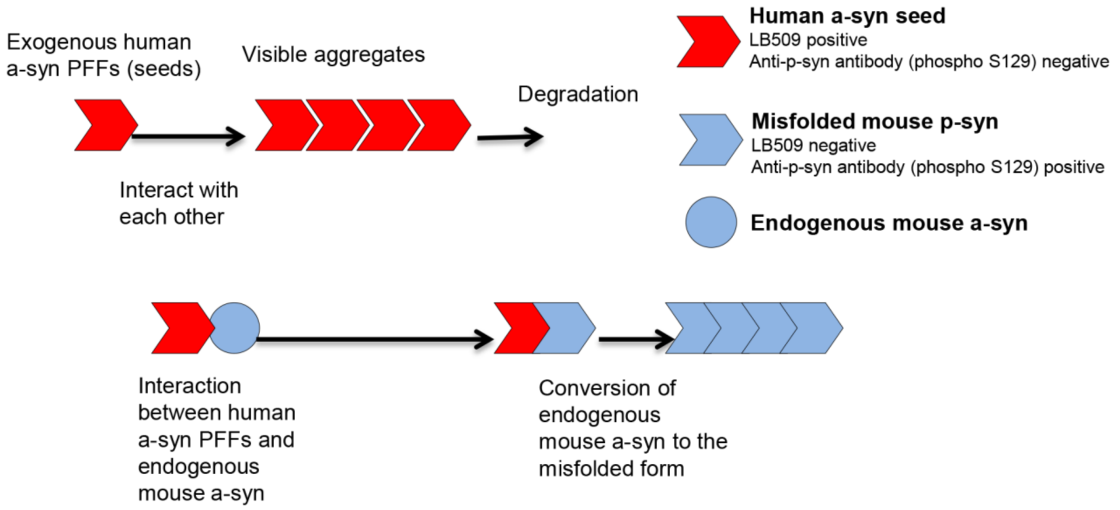

Supplementary Figure S10. Schematic view for the propagation process of endogenous mouse a-syn by exogenous human a-syn seeds

Exogenous human a-syn seeds interact with one another and form visible aggregates, which are not phosphorylated *in vivo*. The part of exogenous human a-syn seeds interact with endogenous mouse a-syn, which may be a small part of endogenous a-syn, and convert them into misfolded forms.

a-syn: alpha-synuclein, PFFs: preformed fibrils, p-syn: phosphorylated a-syn

Supplementary Figure S11.

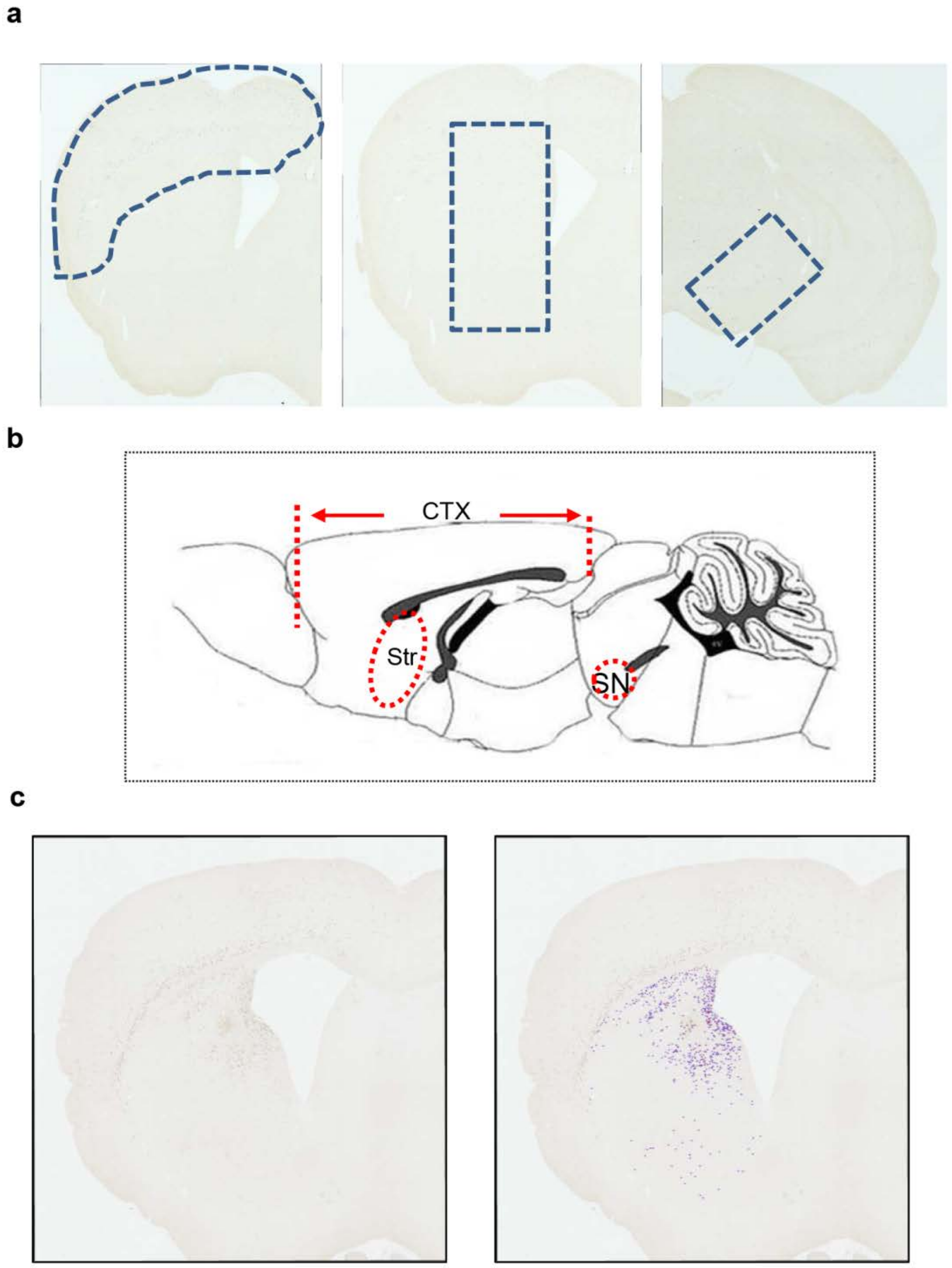

Supplementary Figure S11. Measurement of inclusions (area and number)

(**a**) Whole-brain sections were imaged with a Keyence microscope (BZ-9000) using bright field capture to determine the p-syn inclusions (deposits). For measurement of the inclusion area, continuous sections of the entire brain were

prepared, and the 5-μm-thick sections were selected in sets of 20 (100 μm at a time). (**b**) Cranial to caudal regions of different sites of the brain indicated by dotted lines such as the CTX, Str, and SN were selected. Multiple fields were captured using a 10× objective and stitched together using the Keyence Merge function. P-syn-positive deposits present in every section were selected by the color-extraction mode, and the p-syn inclusions per area were quantified using the BZ-9000 Generation II Analyzer (Keyence) Single Extraction function of the Hybrid Cell Count software based on hue. (**c**) p-syn deposits were extracted using a color extraction method a color extraction method utilizing the BZ-9000 Generation II Analyzer (Keyence). (Left) Before color extraction (Right) After color extraction. Str: striatum, CTX: cortex, SN: substantia nigra, p-syn: phosphorylated alpha-synuclein
